# Approaches to Integrated Parasite Management (IPM) for *Theileria orientalis* with an Emphasis on Immunity

**DOI:** 10.3390/pathogens10091153

**Published:** 2021-09-07

**Authors:** David Lyall Emery

**Affiliations:** Sydney school of Veterinary Science, University of Sydney, Sydney, NSW 2006, Australia; david.emery@sydney.edu.au

**Keywords:** *Theileria orientalis*, *Haemaphysalis*, parasitaemia, integrated parasite control

## Abstract

Integrated parasite management (IPM) for pests, pathogens and parasites involves reducing or breaking transmission to reduce the impact of infection or infestation. For *Theileria orientalis*, the critical impact of infection is the first wave of parasitaemia from the virulent genotypes, Ikeda and Chitose, associated with the sequelae from the development of anaemia. Therefore, current control measures for *T. orientalis* advocate excluding the movement of naïve stock from non-endemic regions into infected areas and controlling the tick *Haemaphysalis* *longicornis*, the final host. In Australia, treatment of established infection is limited to supportive therapy. To update and expand these options, this review examines progress towards prevention and therapy for *T. orientalis*, which are key elements for inclusion in IPM measures to control this parasite.

## 1. Introduction

The fundamental philosophy underpinning the success of integrated parasite management (IPM) for pests, pathogens and parasites involves reducing or breaking transmission to reduce the impact of infection or infestation. For parasites, prerequisites for the rational formulation of comprehensive control measures are: 1, a thorough understanding of the parasite life cycle and mode(s) of transmission; 2, knowledge of the pathogenesis of clinical disease arising from the host–pathogen interaction; and, 3, awareness of the seasonal epidemiology of the parasite–environment interaction that determines fluctuations in parasite populations. For nematode parasites, the various IPM programs comprise 5 major interactive components to reduce parasite availability and infection, thereby reducing pathogenic sequelae and prolonging the effective life of parasiticides by decreasing the need for treatments. From a typical IPM program, “Drenchplan” (https://www.dpi.nsw.gov.au/__data/assets/pdf_file/0004/38551/drenchplan-2005.pdf, (accessed on 27 May 2021)), these components include;

- the effective use of treatments to reduce pathology and parasite reproduction (drenches)
- grazing management to reduce parasite numbers on pasture or prevent host access
- differential management of resistant and susceptible populations (weaning and introductions)
- breeding for parasite resistance to limit parasite numbers in the host
- regular testing to ensure treatments remain effective.

For those parasites with intermediate hosts or vectors, additional control measures target the vector to interrupt or reduce transmission.

Given that *Theileria buffeli*, causing “benign theileriosis” had been present in Queensland, Australia since 1912 [1,2], it was long considered a benign parasite [3,4,5,6,7]. Historically, there was widespread taxonomic confusion regarding various Asian/Australasian theilerial parasites, as *T. sergenti* caused clinical disease Japan and Korea [8]. However, based on morphological and serological data and results from transmission experiments, all members of the *T. sergenti, T. buffeli,* and *T. orientalis* group were classified as a single species, *T. orientalis* [2,5,8]. Phylogenetic analyses using major piroplasm surface protein (MPSP) and p32/34 piroplasmic gene sequences have revealed the genetic diversity of *T. orientalis* in Japan [9,10], Korea [11], Kenya, and Australia [6,12]. Currently, 11 genotypes of *T. orientalis* (type 1 or Chitose, type 2 or Ikeda, type 3 or buffeli, types 4–8, and N1-N3) have been identified based on MPSP gene sequences [10,12]. Of these genotypes, 1 and 2 cause the majority of clinical disease in cattle in Australia [13,14], and elsewhere [5]. These pathogenic genotypes are recognised in many countries including Australia [15], New Zealand [16], Japan [17], Korea [18], and USA [19]. The disease has been estimated to cost the livestock industries in Japan and Korea around USD $100 million annually [17,18]. In Australia, NSW-DPI have estimated an average cost of AUD 59K for dairy producers and AUD 11.6K for beef producers, which equates to AUD 131/head for dairy cattle and AUD 67/head for beef cattle for farms impacted by the parasite, in all, costing around AUD 20m per annum nationally [20]. Mortality rates vary from 1 to 30% in naive stock [20,21].

In the bovine intermediate host, the vast majority of the pathology, clinical disease and deaths results from the anaemia caused by the high levels of parasitaemia associated with the “first wave” of asexual reproduction reaching its maximum around 2–3 months after infection [5,21]. Recovered cattle remain asymptomatic carriers with low levels of parasitaemia (as detected by PCR) for at least 30 months and likely for life [22]. This situation maintains the risk of ongoing tick infestation. Interestingly, several studies from Australia and New Zealand have indicated that the carrier state arising in recovered dairy cattle does not compromise subsequent productivity [23,24]. However, recrudescence of clinical disease may be induced by transport stress, but carrier cattle calve successfully on their home farms. Although mortality remains relatively low in endemic regions, unexposed animals, including calves and introduced stock develop disease around 5–6 weeks after birth or entry [13,22,25]. These cohorts are the focus of theilerial control measures.

Current control measures for *T. orientalis* advocate excluding the movement of naïve stock (from non-endemic regions) into endemic regions while current treatment of clinical theileriosis in Australia is limited to supportive therapy. To update and expand these options, this review examines progress towards prevention and therapy for *T. orientalis*, which are key elements for inclusion in IPM measures to control this parasite.

## 2. Features of the Transmission and Pathogenesis of *T. orientalis*

### 2.1. Life Cycle of T. orientalis

The life cycle of *T. orientalis* begins with the inoculation of sporozoites from the salivary gland of the tick vector (species) as it feeds. After infection as larvae, both nymphal and adult ticks of *Rhipicephalus appendiculatus* and *Haemaphysalis* *longicornis* are capable of injecting sporozoites of *Theileria parva* and *T. orientalis*, respectively [8,26,27]. Sporozoites attach and invade host leucocytes and, following division, become schizonts. These were documented transiently in (parotid) lymph nodes draining tick attachment sites around 7–10 days after infection with Korean isolates of *T. orientalis* in 3/7 calves [8] and 4–8 days after inoculation of tick-derived stabilate [28]. However the schizont stage is not responsible for the pathology associated with the infection, unlike with *T. parva* [29,30]. Asexual development results in uninucleate merozoites, which then escape from parasitised leucocytes and invade erythrocytes where the parasite multiplies into piroplasms. The invasion of red blood cells by merozoites takes place about 10 days post inoculation, and is responsible for the febrile episodes and the clinical manifestations of *T. orientalis*, including the signs associated with anaemia (pale mucous membranes, tachycardia, tachypnoea, weakness) [5,31]. The analogous period with *T. parva* infection is also associated with the most severe inflammatory reactions as leucocytes rupture in submucosal tissues to release microschizonts. Although the mechanism of attachment and invasion of erythrocytes is poorly understood, high parasitaemias can result in severe anaemia and death of cattle [31]. The prepatent period (infection to clinical signs, usually fever) ranges from 7–10 days in Korea [18] and, in Australia, has been variously estimated at 14–47 days [32], 12–16 days after tick application [26,33] to around 20 days after ticks were seen [21]. Therefore, clinical disease occurs well after ticks have engorged (5–7 days) [21], and the prepatent period most likely reflects the quantum of infection as occurs with dose-dependent responses to *T. parva* stabilate [34] and to graded blood volumes of *T. orientalis* Ikeda [35]. In newborn calves and in naïve cattle arriving into endemic zones in Australia, clinical disease associated with anaemia is readily apparent 4–8 weeks after birth or introduction [22,25]. 

Reports indicating that the productivity of carrier cattle appears “normal” [23,24] would suggest that a “steady-state” host–parasite relationship is established after recovery, preventing the recurrence of clinical disease following ongoing, seasonal, tick challenges in endemic regions [6,14,21]. Queensland, the widespread presence of cattle harbouring infections with the “benign” *T. orientalis* buffeli genotype, has been deemed partly responsible for preventing infections with the virulent genotypes in that state [36].

### 2.2. Pathogenesis of Infection with Multiple Genotypes of T. orientalis

In endemic regions with multiple theilerial genotypes present, infections of virulent genotypes Ikeda and Chitose clearly outpace the development of *T. orientalis* buffeli in susceptible cattle [27,37]. In 30 calves sampled from each of 2 Dorrigo farms in 2017 and 2019 where birth dates were available, results confirmed that calves were readily and heavily infected with *T. orientalis* Ikeda and Chitose genotypes within 4–5 weeks of birth [22], similar to results from unexposed cattle introduced into another endemic area in Australia [25]. Weaner cattle introduced to Dorrigo in late summer were PCR-positive within 3 weeks after introduction and exhibited clinical theileriosis within 5–6 weeks after arrival, with an estimated weight loss around 20 kg over the first 3 months [22]. 

#### 2.2.1. Vector Competency, Mechanical and Biological Transmission

The distributions of parasites with indirect life cycles are restricted by the availability of either the intermediate or final host. So the distribution of *T. orientalis* is limited by cattle (the obligate intermediate host) or the definitive host. The full spectrum of biological vectors (or definitive) hosts for *T. orientalis* has not been resolved. Amongst arthropods, the 3-host ixodid tick *Haemaphysalis longicornis* (Neumann, 1901) [38] has been confirmed in transmission trials as one biological vector for *T. orientalis* in early studies [8] in Australia [26,39] and the USA [19]. *H. longicornis* has a predicted, widespread distribution in countries with temperate climates [40], including Australia, New Zealand, Fiji, New Caledonia, China, former USSR, Korea and Japan [41,42], the USA, and several other Pacific Islands, including Hawaii [43].

The vector competency of various 3-host ticks varies across locations, likely related to regional host–parasite adaptations. Uilenberg et al. [8] reported that most global stocks of *T. orientalis* could be transmitted transtadially by *H. longicornis* and *H. punctata*, but not by 3 Amblyomma species or by *Dermacantor reticularis*. However, a USA isolate of *T. orientalis* buffeli was not transmitted by *H. longicornis* ticks from Korea or by *H. punctata* [8,44]. In Japanese investigations, Australian *H. longicornis* could transmit only *T. sergenti* (*T. orientalis* Ikeda/Chitose) and could not transmit *T. orientalis* buffeli, whereas as Japanese *H. longicornis* could transmit both [45]. Early vector studies in Australia indicated that *H. bancrofti* and *H. humerosa* were likely vectors for *T. orientalis* buffeli in northern Australia [33,46]. While *H. longicornis* nymphs and adults readily transmitted *T. orientalis* Ikeda [39,47], these failed to transmit *T. orientalis* buffeli to naïve calves in Sydney trials on 2 occasions [27,47]. This is perhaps not surprising as the definitive distribution of *H.* *longicornis* occurs in the coastal areas of Victoria and New South Wales and extends northwards as far as Gympie in Queensland but is absent from large areas of northern Australia where *Theileria* sp. (*T. orientalis* buffeli) is present [32]. The 1-host tick *Rhipicephalus microplus* was proposed as an alternative vector in India, but while eggs from ticks feeding on infected cattle were positive for *T. orientalis* by PCR, hatched larvae were not tested for any successful transmission [48].

Clinical infection with *T. parva* and *T. orientalis* can be established by tick feeding or through the production and inoculation of a tick-derived stabilate or “GUTS” (ground-up tick supernate) [38,49,50,51]. For the transmission of protozoa, several days of tick feeding is needed to mature sporozoites prior to inoculation into the intermediate host [52]. For the maturation of *T. parva* sporozoites, infected *R. appendiculatus* are fed for 4 days on rabbits [53] to prevent clinical disease if fed on cattle. For *T. orientalis* stabilate production, GUTS produced from newly moulted, adult *H. longicornis* (infected with *T. orientalis* Ikeda as nymphs), were only marginally positive by PCR. Therefore around 3000 of the infected adult ticks were fed for 3 days on an uninfected, splenectomised (SplX) Murray-Grey cross (not *Bos indicus*) before removal and produced strongly positive results in the PCR [39]. Interestingly, while this animal ultimately developed detectable theileriosis it did not develop clinical theileriosis over the subsequent 2.5 months before it was sold. Anecdotal experience at the Tick Fever Centre in Queensland notes that SplX animals can control the initial peak of parasitaemia (up to 10% in blood smears) without reductions in haematocrit and subsequent mortality (P. Rolls, unpublished). The mechanism for these differences with intact cattle is not known. 

Haematophagous insect vectors have also been suggested as involved in mechanical/horizontal transmission, but only the cattle louse, *Linognathus vituli,* has been successfully harvested, transferred, and produced infection in unexposed cattle [54]. Biting flies in large numbers may be another mechanical vector as transfer of a little as 0.1 mL blood was able to establish an infection detectable by PCR [34]. In the field, transmission by lice would appear minimal as the entire life cycle occurs on the host. Mechanical transfer is likely irrelevant to the epidemiology of clinical theileriosis but is only pertinent to the spread of the parasite. As discussed in Section 2.2.2, and for reasons not entirely clear, the mechanical transfer of blood does not appear to cause clinical disease [34]; sexual reproduction in the definitive host (tick) appears necessary to maintain the virulence that is also associated with sporozoite infection. 

Cattle in endemic zones often harbour multiple theilerial genotypes [22,25,55]. *H.* *longicornis* populations feeding on these carriers are also positive by PCR for these genotypes, and multiple genotypes can be detected in tick saliva and stabilate [55]. This indicates that ticks are competent vectors to transmit multiple genotypes to susceptible stock [22]. The intra-genotypic interactions during sexual reproduction in *H. longicornis* leading to cooperation or competition have not been studied. However, recent trials have indicated that *H. longicornis* nymphs, infected as larvae with *T. orientalis* Ikeda, can infect naïve calves and, after moulting, infect additional naïve calves as adult ticks, thereby retaining the original infection across 2 moults [47]. This finding has implications for the control of 3-host ticks and the spread of infection through movement by sylvatic second hosts, such as kangaroos.

#### 2.2.2. Infection with Blood Stages of *T. orientalis*

Successful infection with *T. orientalis*, as detected by smear and PCR, has been consistently achieved by the inoculation of around 10^8^ infected bovine erythrocytes containing several theilerial genotypes [35,47,56,57,58,59]. Both intravenous (iv) and subcutaneous (sc) inocula infected with around 10^8^ *T. orientalis* buffeli or *T. orientalis* Ikeda each produced parasitosis detectable by PCR within 4 weeks [27,47], consistent with previous reports [2,35,59]. In each case, the parasitosis appeared to peak around 6–8 weeks before stabilising at 2000–10,000 gene copies uL^−1^, irrespective of genotype. Infections with 1.25 × 10^7^ parasites had a longer prepatent period [35].

The results from these combined studies indicate that the parasitaemias induced by blood inoculation of single or multiple benign and virulent genotypes of *T. orientalis* do not reach “clinical” levels and do not produce clinical disease in adult cattle or calves >4 months of age [2,35,59]. A similar situation is apparent in calves infected by intrauterine or colostral transmission in endemic zones; these animals remain asymptomatic carriers [47,60,61]. Around 10% of calves born to infected dams were PCR positive at 3 months of age in Japan, but whether clinical disease occurred was not reported [62]. The exact reasons for the differences in pathogenesis between the high parasitaemias generated by tick or stabilate infection and those induced by infected blood remain unresolved.

Since the levels of parasitaemia generated by the inoculation of parasitised blood remain relatively and persistently low and comparable to those in recovered and carrier cattle [22], the method could be examined for immunization against tick challenge (see Section 5.3 below). 

## 3. IPM by Vector Control

### 3.1. Chemical Control of H. longicornis

With confirmation of the definitive host, chemical trials targeting *H. longicornis* are underway, but most results remain unpublished. In Australia, historical effort has focused on treatments for the 1-host ticks, *R. microplus* and *R. australis,* to control bovine babesiosis, while similar regimes have been developed to control the 3-host tick *R. appendiculatus* [63]. Flumethrin pour-ons have reduced numbers of *H. longicornis* and theilerial infections in Korea, with tick control remaining the main focus for control of the infection [64]. Macrocyclic lactones (MLs; moxidectin) have been reported to provide partial reduction in tick numbers on cattle, but infection is still readily transmitted in endemic regions (C. Shirley, unpublished). Three-host ixodid ticks are more difficult to control than 1-host ticks, as these only feed 5–7 days to engorge and are not host-specific [39,65]. However, since around 3 days are required to mature sporozoites, the rapid 12 h knockdown (“speed of kill”) provided by isoxazoline acaricides against ixodid ticks, including *H. longicornis,* on companion animals [66,67] would be ideal to prevent transmission if developed for livestock (provided that the residue limits are acceptable) [65]. As noted in Korea and observations in Australia where reduced tick numbers result in decreased clinical disease [64,68], vector control may reduce the number of sporozoites inoculated, enabling infected cattle to control the intensity of the ensuing parasitism. 

To limit the spread of ticks on purchased cattle, acaricide treatment prior to transport would be required and noted on vendor declarations. However, the movement of ticks outside of their current distributions may not guarantee their continued survival.

### 3.2. Vaccination against H. longcornis

During blood feeding on immunized animals, haematophagous parasites also ingest antibodies which may target their gut antigens, digestive enzymes, or microflora. Successful vaccines against *R. australis* (formerly *Boophilus microplus*) with Tickguard [69] and the nematodes *Haemonchus contortus* (Barberva) [70,71] and hookworms [72] have prompted ongoing developments and vaccination strategies using gut antigens.

There are no successful vaccines currently available for *H. longicornis* in cattle despite the isolation of several candidate antigens, including proteases, ferritins [73], and subolesin [74]. New technological advances in tick genomics, transcriptomics, and microbiome analysis offer the possibilities to target endosymbionts [75,76] or crucial genera in the gut microbiome of *H. longicornis* to reduce reproductive capability or to block the maturation and transmission of parasites [77,78]. Given that *H. longicornis* is parthenogenic, it is not known whether doxycycline or tetracycline targeting endosymbionts might compromise tick development and reproductive fitness [79].

## 4. IPM through Chemotherapy to Prevent Development and Persistence of *T. orientalis*

While effective acaracides prevent the development of pathogenic theilerial genera in the final host, the control of theilerial species in the intermediate can target schizont development or remove blood stages that could be ingested by the final tick host. In prevention or retarding the development of *T. parva* in the “infect and treat” protocol, oxytetracycline and stabilate are administered concurrently [50,51]. Due to the difficulties in producing tick-derived stabilate, this has not been attempted for *T. orientalis*, but anecdotal evidence suggested that toltrazuril may attenuate schizont development in calves (C. Shirley, unpublished).

### 4.1. Prevention of Parasite Development in the Intermediate Host

Toltrazuril (Baycox) is known to be active against the schizont stages of Eimeria and Isospora spp., which are related to Theileria [80]. Baycox remains at therapeutic levels in calves for around 8 days [81], which would “cover” the early schizont development. However, when administered at 15 mg/kg to 20 dairy calves, 4 weeks after turnout in New Zealand, Baycox did not prevent or ameliorate theilerial parasitaemias significantly [82]. Similarly in Australia, Baycox was given at 15mg/kg, to 15 calves, 4 days following challenge with 50 unfed adult *H. longicornis* that had been infected as nymphs with *T. orientalis* Ikeda. The timing was specifically aimed to coincide with the early schizont stages of the parasite and included the 3 days of feeding required to mature sporozoites in *H. longicornis* prior to inoculation [39]. In comparison with infected but untreated calves, toltrazuril had no significant effect on developing parasitaemia [83]. At this time, oxytetracycline has not been examined for “infect and treat” regimens for *T. orientalis*.

### 4.2. Chemotherapy of the Carrier State

Another means to break the transmission of theilerial parasites in endemic regions is to cure the carrier state. Experimentally, this develops after the mechanical transfer of infected blood and in the field, after the first wave of parasitaemia. Several compounds were utilized for a chemotherapeutic trial with selections based on the premise that clinical disease from *T. orientalis* coincided with the appearance of piroplasms, fever, and parasitaemia, and that these merozoite stages multipled in erythrocytes like babesial and malarial parasites. So drugs with activity against other haemoprotozoa with important erythrocytic stages for asexual development could be effective against *T. orientalis*. In Queensland, *T. orientalis* buffeli could be cured by the administration of primaquine and halofuginone or primiquin and buparvaquone (BPQ) [84,85].

Previously, oxytetracycline and imidocarb (Imadox) have been used for the “treatment” of clinical cases of *T. orientalis*. The napthoquinones, parvaquone and BPQ, and the febrifuginone, halofuginone lactate, will cure clinical disease associated with *T. annulata* or *T. parva* [86,87] but are not registered for clinical use in Australia. BPQ targets the schizont stage of the parasite, which is associated with the clinical signs in East Coast fever (ECF). BPQ also reduces number of *T. orientalis* Ikeda piroplasms in blood within 4 days [88,89], while the addition of chloroquine, quinine, or pyrimethamine to bovine blood cultures in vitro inhibited the proliferation of *T. orientalis* [sergenti] [90].

When 4 of these potential therapeutic compounds were examined for effects on blood-induced infections with *T. orientalis* Ikeda, only BPQ suppressed parasitosis; imidocarb (Imadox), tulathromycin (Draxxin) and oxytetracycline had no effect ([27]; S. de Burgh, unpublished). However, BPQ did not cure the infection as recipients still were PCR-positive 2 months later. Further field work would be needed to confirm BPQ’s effectiveness in clinical outbreaks, but it would not contribute to IPM programs. This study discounted the use of oxytetracycline and Imadox for the treatment of *T. orientalis*, while Imadox also failed to reduce parasitaemias (detected in blood smears) in 3 calves infected with *T. orientalis* (Ikeda (P. Carter, unpublished).

It is possible that further testing may reveal more effective compounds, but the low mortality rates from *T. orientalis* may not justify investment. Drugs used for the treatment of canine babesiosis or human malaria (not registered for use in cattle anywhere) are expensive, lack residue depletion data, and likely to have the same lack of efficacy. Diminazene (Berenil) and primiquin [91] are still possibilities to examine, the latter (pamaquin and primaquine) being active against the piroplasms of *T. annulata* [92,93] but are unable to cure *T. orientalis* buffeli parasitosis [2]. However, due to residues and withholding periods, these appear less important in the overall integrated management of clinical theileriosis, and the parasiticide is often administered too late if clinical signs are already apparent. Parenthetically, this “timing issue” gives rise to anecdotal “cures” for *T. orientalis*, when compounds are administered after animals have passed the first peak of parasitaemia and have entered the recovery phase towards carrier status.

In endemic regions with ongoing seasonal tick challenge, the carrier state appears to prevent the recurrence of clinical theileriosis without reducing productivity, so that curing the carrier state may not be beneficial.

## 5. IPM to Produce Resistant Hosts: Immunisation against Infection

The fourth significant component for theilerial IPM is the generation of resistant livestock. Apart from tick resistance between cattle breeds [94], innate genetic resistance to theilerial parasites appears to be lacking, and vaccination provides an alternative approach. The precise mechanism providing protective immunity against *T. orientalis* remains unresolved. Recovered animals enter a persistent carrier state after the “first wave” of parasitosis with the virulent genotypes of *T. orientalis* around 2–3 months after infection, whether from tick infestation or stabilate containing single or multiple theilerial genotypes [22,23,35,37]. This situation also reflects field experience wherein recovered cattle may harbour multiple theilerial genotypes in the carrier state [13,22,49]. In recovered cattle, some type and level of immunity exists in carrier cattle which resembles a “premunity” [95], interfering with the severity of subsequent challenge infestations [2]. The use of premunity has a long history in early “vaccinations” against Leishmania, malaria, East Coast fever, babesiosis and poultry coccidiosis (“precocious strains”) see [96,97]. In Australia, pre-existing infections with *T. orientalis* buffeli actually suppressed infections with *B. bovis* and *Anaplasma marginale* but not *B. bigemina* in SPLx calves [98] and could do so for *T. orientalis* Ikeda in an experimental trial [47].

Passive infection during gestation or through colostral antibodies do not appear to provide reliable protection against *T. orientalis*. In Korea, intra-uterine infection with *T. orientalis* [sergenti] occurred readily, but did not protect against field challenge after birth [60,61]. The same situation occurs in endemic regions of *T. orientalis* in Australia [22]. Calves born of infected dams are not fully protected against tick challenge with *T. orientalis*, indicating that colostral antibodies are not protective [60,61]. The effect may be due to genetic diversity in genotypes of *T. orientalis* or in their MPSP genes (and epitopes) [20,99]. The lack of protection may also reflect the low “immunizing dose” in utero or from colostrum, or the level of tick challenge, as parasitaemia following infection with *T. orientalis* Ikeda by 200 *H. longicornis* nymphs was significantly reduced in two calves thT were presumably infected during gestation or at birth with the Ikeda genotype [47].

### 5.1. Immunisation against Theilerial Parasites (T. parva, T. annulata)

Since the principal protective immune response against *T. parva* is genetically restricted CD8+ -mediated lympho-cytotoxicity (CML) against the macroschizont-infected lymphocyte [100,101], protection requires the live parasite for induction but is restricted to the parasite genotype in the vaccine. This underscores the effectiveness of stabilates containing sporozoites of *T. parva* to generate protection against challenge when administered with long-acting oxytetracycline in the “infect and treat” method [50,51]. However, as predicted, limited cross-protection is produced, particularly against buffalo genotypes, and multivalent stabilates can lead to the recombination of *T. parva* genotypes during the immunization period [101]. A similar restricted protection is induced by the administration of around 10^8^ allogenic cultured lymphoblasts infected with macroschizonts of *T. parva* [100] or 5 × 10^6^ cells infected with *T. annulata* [102]; reinforcing the importance of this parasitic stage to the pathology and protective immunity against both parasites [103]. The lack of cross-protection and complications from the exquisite specificity of CML has fostered research into other antigens and modes of delivery [101,104].

As discussed above, the merozoites/piroplasmic stages of *T. orientalis* appear more important than the schizont to the pathogenesis and anaemia of clinical theileriosis [5]. In field trials, GUTS stabilate for *T. parva* immunizations equate to around 10 ticks per dose [105], whereas stabilate used for *T. orientalis* infection was around 30 ticks per dose [39]. The lower infection rates in *H. longicornis* and reduced mortalities compared to *T. parva* do not justify pursuit of an “infect and treat” protocol for *T. orientalis*, so alternative strategies have been investigated.

### 5.2. Immunisation against T. orientalis with Inactivated or Subunit Vaccines

To avoid the possible transfer of pathogens using whole blood vaccines, the search for protective antigens and vaccine formulations from the blood stages of *T. orientalis* have been based on three principal research outcomes. Firstly, these stages of the parasites are deemed responsible for the clinical sequalae of the infection, and, secondly, the molecular typing of the MPSP has clearly identified the virulent genotypes [5,10]. Moreover, the temporal kinetics for genotypes of *T. orientalis* during ongoing infections has been attributed to “escape” from protective, MPSP-specific antibodies [37,97]. This resembles the sequential production of neutralizing antibodies to variant-specific surface antigens (VSSA) of trypanosomes, although in this infection, the gene splicing by the parasites produces novel VSSAs ahead of the host response [106].

Further support for subunit vaccines had been encouraged from several trials attempting to identify candidate antigens, especially involving MPSP, to generate neutralizing antibodies for genotype-specific protection. The passive transfer of ascitic fluid from hybridomas recognising the P32 protein from *T. orientalis* [sergenti] prevented the development of parasitaemia in 3 SplX calves challenged with *T. orientalis* Chitose merozoites from infected blood [107]. However, this was not tested against tick challenge or sporozoite-based stabilate. In immunization trials using dissociated parasites, calves were inoculated with 2 × 100 mg doses of sonicated *T. orientalis* [sergenti] merozoites in complete Freund’s adjuvant subcutaneously. When subjected to field challenge 2–5 months after the initial vaccination, their parasite burdens were significantly reduced at 3 months post-challenge, [59]. Unfortunately, this trial was terminated 2 months later as all controls and 20% (4/20) of vaccinates required treatment with diminazene (Berenil) for anaemia [59]. The outcome would indicate that, while the vaccine had induced substantial protection, either the field challenge overwhelmed (a waning) immunity or field strains contained virulent genotypes that were not present in the vaccine.

Two studies also investigated the efficacy of recombinant MPSP in vaccines. Calves immunized with recombinant MPSP from “I” (Ikeda) and “C” (Chitose) genotypes in Freund’s adjuvant or liposomes showed “vaccine effects” after challenge with a stabilate containing both “variants” (genotypes) [57]. This study was the first to indicate that cross-protective immunity could be generated against genotypes of *T. orientalis*, but the levels of parasitaemia were not reported. A recombinant MPSP vaccine for *T. orientalis* [sergenti] utilised three vaccinations at 3-week intervals, producing an antibody response but no protection against challenge [108].

From the lack of consistent generation of protective immunity, these recombinant vaccines have not been pursued to date.

### 5.3. Immunisation against T. orientalis with Blood Stabilates

Reports from field infections in endemic regions of *T. orientalis* consistently indicate that recovered carrier cattle resist seasonal reinfection from ticks [20], and ongoing productivity appears to be unaffected [23,25]. Since the blood stages of *T. orientalis* cause the clinical pathology and any untoward deaths, it is not surprising that blood-based vaccines have been examined, with variable success [5,36]. Historically, a blood vaccine containing 2 × 10^8^ infected red blood cells per dose “had an inhibitory effect on the clinical manifestation of *T. orientalis* [sergenti]” with a need for “proliferation of the inoculum” [56,60] but this was not continued. A whole blood vaccine against *T. orientalis* [sergenti] was tested in Korea but outcomes were not reported and challenge appeared to use blood stabilate (see [58]). Inocula of blood containing live *T. sergenti* induced variable levels of protection against tick challenge in Japan, an effect attributed to genetic variations in the MPSP p32 protein across the country [57].

Dose-response studies on blood vaccines have not been completed due to the rather ad hoc history of these trials [36], but several results emphasise that dose may influence the levels of protection generated. Both the passive infection in utero and the transfer of antibody does not appear to provide protective immunity after birth [60,61], and this may be related to low dose infection and the transient time-frame of maternal antibody under consistent tick challenge (Section 4 above). However, the interval between (passive) infection and tick challenge, the intensity of the tick challenge, and nutrition may affect the outcome, with calves consistently parasitized by 3–4 weeks of age [22]. Another complication for vaccines against *T. orientalis* is possible genetic diversity within genotypes [10,109], a problem already appreciated for *T. parva* [101].

Whole blood vaccines against *T. orientalis* were not pursued due to the possible transfer of viruses [58]. More recently, we have revisited the blood vaccine approach with the “benign” buffeli genotype [36], as few cases of clinical theileriosis occur in Queensland where this genotype has been present since 1910 [3]. However, as mentioned previously, competent tick vectors for *T. orientalis* buffeli and the virulent genotypes are different in Australia [33,46]. Calves inoculated intravenously (IV) or subcutaneously (SC) with blood infected with *T. orientalis* buffeli became PCR-positive within 4 weeks. The infection was allowed to “mature” for another 2–10 weeks. When challenged with 200 nymphal *H. longicornis* infected with the Ikeda genotype of *T. orientalis*, the first peak of parasitemia was significantly reduced by up to 80% between 6 and 9 weeks after challenge [27,47].

This mode of protection would not work for calves in endemic zones as the virulent genotypes appear first [22,25]. It would be possible for proposed introductions that could be “immunised” before movement into endemic areas. Consequently, groups of 12 cattle aged 8–10 months were inoculated SC with either *T. orientalis* buffeli or Ikeda and were positive 4 weeks later; a control group remained negative. Six weeks after “immunization”, 35 animals were transported 700 km to an endemic region with “heavy” tick challenge. Inoculated animals did not recrudesce with clinical theileriosis, and no animals died during the first 6 months after arrival in spring. Those given *T. orientalis* Ikeda had significantly reduced parasitaemia during the first wave 6–9 weeks after arrival. In contrast to the previous study, prior immunization with *T. orientalis* buffeli was not significantly protective (D. Emery, unpublished). PCV and weight gains showed a similar effect, but with adequate feed available over summer, the initial weight losses had been recouped by compensatory growth within 6 months after introduction (D. Emery, unpublished). The different outcomes with *T. orientalis* buffeli was likely due to the heavier tick challenge, and studies are ongoing.

Field trials in endemic regions with high levels of tick infestation carrying multiple genotypes is vital to determine the robustness of this method to reduce the impact of *T. orientalis* on survival and productivity. Despite the difficulties of experimental tick infestation, these may be required to determine dose rates and genotypic combinations for establishing any reliable immunisation protocols. There may also be some synergy for a combination of “immunisation” before movement and the application of long-acting effective acaricides on arrival to attenuate the tick challenge by also reducing the quantum of ticks infesting immigrant cattle. However, once entering the carrier state, productivity is expected to attain normal benchmarks [23,25]. Currently, restricting the levels of tick infestation is the most viable option for the prevention of clinical theileriosis in newborn calves in endemic regions.

## 6. Conclusions and Further Research

The cattle industries in regions of endemic *T. orientalis* would benefit substantially from measures to reduce the impact of the initial infection either by means to control the vector or the early stages of the pathogenesis of the infection. If this can be managed into the carrier state, then animals under conventional husbandry appear to be protected from further clinical disease.

For protection of cattle moving into endemic zones, the deliberate pre-infection of cattle prior to movement requires further field trials in endemic regions with high levels of tick infestation carrying multiple genotypes are needed to determine the robustness of the procedure. Despite the difficulties of experimental tick infestation, these may be required to determine dose rates and genotypic combinations for establishing any reliable immunisation protocols. There may also be some synergy for a combination of pre-infection before movement and the application of long-acting effective acaricides on arrival to attenuate the tick challenge by also reducing the quantum of ticks infesting immigrant cattle [110,111], combined with rotational grazing after arrival [112].

However, pre-infection with merozoites does not address the other at-risk cohort; neonatal calves born in endemic regions of *T. orientalis.* For these animals, tick attachment and infection with *T. orientalis* Ikeda and Chitose occurs within the first week after birth. Limiting tick numbers could be approached by the location of calving paddocks well removed from bushland to avoid *H. longicornis* carried by wildlife or possible movement of calving times to avoid the seasonal appearances of adult ticks around spring. Since calving intervals are usually 6–8 weeks duration, producers are reluctant to muster animals for acaricide treatment of neonatal calves. However, the judicious use of effective existing and new acaricides offer additional control options to reduce tick numbers and limit the dose of theilerial genotypes transmitted for neonatal calves and introduced cattle.
